# Nurses’ unprofessional behavior on patient care—A scoping review

**DOI:** 10.1177/09697330261441782

**Published:** 2026-04-10

**Authors:** Satu Lyytikäinen, Mari Kangasniemi, Tanja Moilanen

**Affiliations:** 1 Department of Nursing Science, Faculty of Medicine, 8058 University of Turku, Kuopio, Finland; 2 The Wellbeing Services County of Southwest Finland, Turku University Hospital, Kuopio, Finland; 3 Wellbeing Research Field, 4345 Savonia University of Applied Sciences, Kuopio, Finland

**Keywords:** nurses, patient safety, scoping review, unethical behavior, misconduct, unprofessional behaviour

## Abstract

**Background:**

Nurses’ unprofessional behavior endangers quality of care and patient safety as well as nurses’ careers and work communities. Existing knowledge concerning nurses’ unprofessional behavior and its effects on patient care should therefore be considered to facilitate early identification and the development of preventive strategies.

**Aim:**

The aim of this scoping review is to systematically map and summarize existing knowledge about unprofessional behavior among nurses in order to support the development of strategies for its early detection and prevention.

**Method:**

A scoping review was performed based on electronic searches of the CINAHL, PubMed, Scopus, Web of Science, and ERIC databases together with manual searches of the reference lists of all publications retrieved in the initial searches. The database searches spanned the period from database creation to October 2024. Only peer-reviewed empirical research papers published in English were considered for inclusion. Publications were selected using predefined inclusion and exclusion criteria, and altogether 40 studies were included. Data was analyzed using descriptive methods.

**Findings:**

The terms used in relation to unprofessional behavior are related to neglect and abuse, unethical behavior, and misbehavior. Types of unprofessional nursing behavior during patient care include neglecting nursing tasks, betraying professional confidence, and violating patient integrity. These behaviors may create risks to patient safety and cause patient dissatisfaction. Reasons for unprofessional behavior included nurses’ personal life histories, lack of professional competencies, and factors relating to the work environment and community.

**Conclusions:**

Our findings indicate that unprofessional behavior by nurses is an umbrella term, covering various forms of inadequate and unethical behavior. In future, more attention must be paid to nurses’ working conditions and ethical skills to protect patients’ safety and strengthen the nursing profession.

## Introduction

Patient safety is an essential part of high-quality care but may be threatened by unprofessional behavior on the part of nurses.^1^ Nurses are the largest group of healthcare professionals and are thus involved in a majority of patient contacts. Consequently, they play a key role in ensuring ethical care. However, unprofessional behavior by nurses is a global concern.^2–6^ Approximately one-third of all nurses have experienced unprofessional behavior by their co-workers^7,8^ and almost three in four have experienced or witnessed peers violating boundaries.^9,10^ Growing concerns about unprofessional behavior have prompted exploration of this phenomenon from various perspectives to facilitate its early recognition and detection.^2,3,8,11–13^

Nurses’ unprofessional behavior refers to different types and varying degrees of negative behavior. Unprofessional behavior includes incivility^14–16^ and disruptive behavior in the workplace,^17^ actions that violate boundaries^18^ as well as violent and inappropriate behavior towards patients,^18–20^ colleagues,^14,17–19,21^ and/or the wider healthcare organization.^1,21,22^ Unethical behavior of nurses can be defined as a behavior that brings harm to another person or is illegal or violates patient’s rights.^2^ Professional misconduct refers to behavior that is unethical, dishonest, and violates rules and regulations. Misconduct can have legal consequences for professionals, who may lose the public trust and confidence.^23^

There is evidence that unprofessional behavior by nurses during patient care is partly due to the demands of the current healthcare situation, which is characterized by stressful work environments and overloaded workers. Nurses are regularly faced with complex situations during their work and must meet many demands to provide optimal care, which can be overwhelming and may create ethically challenging situations or ethical dilemmas, leading to stress.^5,6^ Exhaustion caused by dissatisfaction with organizational practices and management of human resources can also lead to unprofessional behavior.^14,16^ Unprofessional behavior by nurses during patient care has caused adverse events and errors,^17,21^ jeopardizing patient safety^21,22^ and endangering staff wellbeing.^7,8^

There is also evidence that intervening to prevent unprofessional behavior by nurses can be challenging. Unprofessional behavior often occurs between a nurse and a patient in situations where no witnesses are present^18^ and the patient is in a vulnerable position.^19,24^ In addition, peer pressure and loyalty to colleagues may discourage other nurses from whistleblowing on such situations.^11,25^ Overall, then, the phenomenon of unprofessional behavior by nurses is multidimensional, only vaguely defined at present, and not always straightforward to identify.^11,22^ Systematic mapping of current knowledge is therefore needed to identify the various types of unprofessional nursing behavior that have been reported to facilitate its identification and prevention in clinical practice.^26^

## Aim

The aim of this scoping review is to systematically map and summarize current knowledge about unprofessional behavior by nurses, in order to guide the development of strategies for early detection and prevention of unprofessional behavior in the future.

The research questions were as follows:

(1) What terms have been used to describe nurses’ unprofessional behavior?
(2) What types of unprofessional behavior by nurses during patient care have been described?
(3) What are the consequences of nurses’ unprofessional behavior for patient care?
(4) What reasons for unprofessional behavior during patient care have been identified?

## Methods

We conducted a scoping review to explore the full breadth of unprofessional behavior by nurses.^26,27^ The review process had five steps: (1) identifying the research questions, (2) identifying potentially relevant studies, (3) selecting studies, (4) charting, and (5) collating, summarizing, and reporting the data.^26,28^

### Identifying the research questions

The research questions were set after acquiring familiarity with the literature by performing preliminary database searches.^26^

### Identifying relevant studies

**Figure 1.**
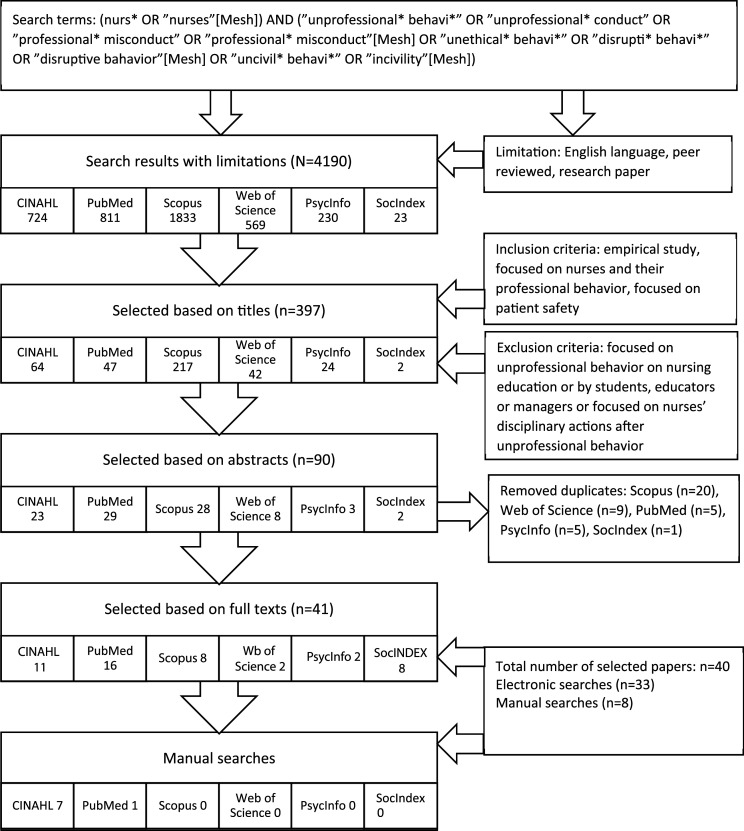
Flowchart showing the systematic literature search protocol and data selection process.

Potentially relevant studies were identified^26^ by performing electronic searches of the CINAHL, PubMed, Scopus, Web of Science, and ERIC databases. The search terms were words relating to nurses and unprofessional behavior (Figure 1). The searches were limited to peer-reviewed empirical research papers published in English. The searches were limited to articles published between their appearance of the database and October 2024. Manual searches were performed on the reference lists of selected articles. An informatician working at a university library was consulted to validate the search strategy and the relevance of the search terms.

### Study selection

Study selection was guided by a set of inclusion and exclusion criteria^26^ relating to the studied population and the relevance of the study’s terms and context to the research questions. The inclusion criteria were studies focused on (1) nurses, (2) unprofessional behavior, and (3) patient care. Studies were excluded if they focused on (1) nurses’ unprofessional behavior during nursing education; (2) unprofessional behavior by students, educators, or managers; or (3) disciplinary actions taken against nurses after unprofessional behavior.

Papers were selected based on their titles, abstracts, and full texts. Selection was performed by three researchers working collaboratively SL, MK, TM to minimize selection bias. Disagreements about the inclusion or exclusion of studies were resolved by discussion until consensus was reached.

### Charting, collating, and summarizing the data

The fourth stage was charting the data. First the entire data was read to get an overview of its content.^26^ Next, the papers were tabulated by author(s), year, country, aim(s), methods, main results, and terms applied (Appendix 1).

The fifth data analysis stage involved collating and summarizing the data^26^ using the descriptive analysis method.^28^ Each selected publication was read repeatedly to get a deep understanding of its content. Descriptive expressions concerning unprofessional behavior by nurses, terms and types used in previous studies, consequences for patient care, and reasons for unprofessional behavior were then extracted. An expression could be as short as a single word or as long as a phrase or paragraph. The expressions were first grouped based on their similarities and differences, and then categorized and organized based on their content. Finally, they were summarized, described, and visualized in tables. All data charting was initially performed by one researcher and then reviewed by the entire research team.

### Findings

#### Description of the studies

Forty studies satisfied the inclusion criteria and were included in the data. Twenty-three of the studies were qualitative, of which twenty-two were qualitative interview studies, two qualitative questionnaires based, and one observation study. Fourteen quantitative studies were included, of which eleven were surveys and three were document analyses. Three studies used mixed methods with quantitative and qualitative surveys or document analyses. Unprofessional behavior by nurses and its impact on patient care were investigated in special medical care departments and emergency departments (*n* = 20), in delivery wards and midwifery (*n* = 14), in mental care (*n* = 2), in elder care (*n* = 2), and at a center for homeless people (*n* = 1). In addition, four studies investigated reports concerning allegations made against nurses. Thirty studies were conducted from the perspective of nurses or other healthcare professionals, and eleven included the patients’ perspective. The informants in the studies were registered nurses or nurse assistants (*n* = 9675), other health professionals (*n* = 3317), patients (*n* = 1232), physicians (*n* = 613), nursing students (*n* = 185), nurse managers (*n* = 105), midwives (*n* = 275), and spouses of delivering mothers (*n* = 6). The studies were conducted in North America (*n* = 12), European countries (*n* = 9), Africa (*n* = 8), Asia (*n* = 7), and Australia (*n* = 4).

#### Terms used in previous studies

Unprofessional behavior has been described in the literature using several different terms (*n* = 13) relating to neglect and abuse, unprofessional conduct, and misbehavior (Table 1).

**Table 1.** Terms used to describe unprofessional behavior by nurses in patient care.

Groupings of related terms	Terms used in the included studies
Terms related to neglect and abuse	Disrespectful and abusive care^9,28–32^
	Mistreatment and abuse^33–37^
	Care-related abuse and neglect^38^
	Inadequate care and neglect^39^
Terms related to unethical behavior	Unethical behavior^6,40^
	Unethical conduct^12,41^
	Moral disengagement^42^
Terms related to misbehavior	Professional misconduct^8,43–49^
	Unprofessional conduct/behavior^50–52^
	Sexual misconduct^53,54^
	Disruptive behavior^55–59^
	Incivility^60–62^

##### Terms related to neglect and abuse

Disrespectful and abusive care^9,19,29–32^ and mistreatment and abuse^33–38^ were used to describe displays of verbal and physical violence towards mothers in childbirth. Care-related abuse and inadequate care and neglect referred to disrespectful behavior, disregard of patients’ needs, and false treatment in long-term care.^39,40^

##### Unethical behavior related terms

Terms used to describe unethical behavior by nurses included unethical behavior,^6,41^ unethical conduct,^12,42^ and moral disengagement.^43^ Compromised ethical values were linked to physical and psychological maltreatment of patients, discrimination, and ignoring personal boundaries.^41^ Unethical behavior also included careless handling of nursing duties,^43^ dishonesty, unaccountability,^42^ and negative behavior towards peers.^12^

##### Misbehavior related terms

The terms of incompetence and impairment were used in relation to professional misconduct and poor general care by nurses.^44–46^ Terms used in relation to allegations of errors or poor job performance by nurses included unprofessional conduct and professional misconduct.^8,47–52^ Violations of professional boundaries by nurses included sexual misconduct^44,53,54^ and opioid addiction.^55^ Inappropriate behavior towards colleagues in the workplace was described using the terms of disruptive behavior and incivility^56–60^; such behaviors have negative effects on teamwork, job performance, exposure to adverse events, and patient safety.

### Types of unprofessional nursing behaviors reported during patient care

Reported examples of unprofessional behavior by nurses during patient care include neglect of nursing duties, betrayal of professional confidence, and violations of patient integrity (Table 2).

**Table 2.** Types of unprofessional behavior by nurses during patient care.

Main category	Sub-category	Group
Neglect nursing tasks	Treatment failure	Incorrect treatment^6,8,22,32,39,40,48,49,52,59,61^
		Neglect treatment^8,9,12,30–41,43,48,50,56,59,61–64^
	Information failure	Poor reporting and documentation^8,22,42,46,47,50,56,57,59,61,62,64^
		Deficient communication^8,30,33–38,41,47,48,65^
Betrayal of professional confidence	Unreliability	Use power against patient^6,9,30,31,33–36,39–41,43,48,62^
		Break patient`s confidence^6,33,39,41,52^
	Rule braking	Dishonesty and stealing^8,22,39,40,42,46^
		Bending rules^8,48,51,52^
		Substance use^39,44–46,55^
Violations of patient integrity	Physical misbehavior	Aggressive and violent behavior^9,31–33,35,37,38,41,47,48,62,65^
		Intentional harm to patient^33,35,39,49,62^
		Unnecessary examinations^30,33,41,48^
		Unnecessary restrain patient^30,36,39,40,43^
	Psychological misbehavior	Uncompassionate behavior^30,33–38,47,48,52,61,62^
		Disrespectful behavior^9,30,33,35,38,40,41,47,48^
		Verbal abuse^9,30,33,34,36–39,42,44,62,65^
		Intimidating behavior^9,30,33,35,36,39,40,48,61^
		Discrimination^33,35–38,42,47,48,61,62^
	Sexual misbehavior	Violation of personal boundaries^39,44,46,54,65^
		Sexual abuse^39,44,47,54^
		Inappropriate relationship with patient^44,46,54^

#### Neglect of nursing tasks

Neglecting nursing tasks has been a type of unprofessional behavior by nurses that encompasses failures of treatment and information such as applying incorrect treatments, neglecting treatment, poor reporting and documentation, and deficient communication (Table 2). Incorrect treatment by nurses^22,39,48,59^ has included failing to display necessary competencies in assessing or clinically managing patients and breaking rules pertaining to standards of care.^6,8,32,40,48,49,52,61^ Nurses neglecting treatment has included instances of ignoring patients’ needs, leaving patients without essential care,^9,36,39,40,48^ and not providing timely care.^35,36,38,43,59,61–63^ There have also been refusals to aid colleagues and delays in treatment,^12,56,63^ as well as instances of nurses ignoring patients’ pain or distress^30–32,48,62^ and leaving patients unattended^33–35,39^ when needing critical care^9,30,36–38^ or in uncomfortable and unsafe conditions.^8,30^ In addition, it have included failing to advocate for patients^41,64^ and leaving patients without supportive care.^37,48,64^ Poor teaching of novice colleagues has resulted in the creation of inadequate clinical and patient safety protocols.^48,50^

Unprofessional nursing behavior in relation to patient care has referred to poor reporting to colleagues or deliberately withholding important information.^8,50,56,57,59,62,64^ It have also involved failing to acquire sufficient background information about patients,^62^ and documenting procedures incompletely or inappropriately documenting procedures that have not been performed,^46,47,61,62^ or hiding the truth about care events.^22,42^ Deficient communication on the part of nurses has involved adopting a disrespectful and rude attitude towards patients^30,33,36,48,65^ and not preparing adequately for procedures.^8,30^ Other nurses have left patients uninformed about their health and treatments^30,33–37,41,47,48^ and failed to explain their actions.^36–38,48^

#### Betrayal of professional confidence

Unprofessional nursing behavior falling into the betrayal of professional confidence has reported to lead to the loss of patients’ confidence in the care relationship (Table 2). This has included being unreliable and breaking rules, by being dishonest or using power against patients and thus breaking their confidence. It also has included stealing, bending rules, and substance abuse at work. Some nurses have taken advantage of their position and used power against patient by acting without patient consent, forcing patients to do things against their will,^9,30,33,35,36,39,48,62^ and preventing patients from making decisions about their own care.^6,31^ Patients have been prohibited from using alarms^40,43^ or items they need,^9,35,36,39^ violated patients’ physical and psychological privacy^6,9,30,31,41,62^ by exposing them to others,^33,34,39,41,62^ entering rooms without knocking,^40^ and refusing to leave the patient’s room when asked to do so.^39^ Nurses have broke patients’ confidence by sharing information about their medical condition or personal life with outsiders^33,39,41,52^ or family members but not with the patient.^6^

Betrayal of professional confidence by nurses has involved dishonesty and stealing.^8,22,39,40,42^ There have been cases where nurses have intentionally defrauded and taken financial advantage of vulnerable people.^42,46^ Nurses have betrayed their professional confidence by bending the rules of care standards and ignoring patients’ best interest.^8,48,51,52^ Unprofessional behavior by nurses has involved abusing substances during working hours by themselves^39,45,46,55^ or with patients.^44^ In addition, some nurses have stolen drugs from the workplace for their own use, causing patients to suffer for want of pain alleviating medicines.^55^

#### Violations of patient integrity

Reported violations of patient integrity by nurses have referred to actions that undermine a patient’s dignity, privacy, autonomy, or bodily respect within in a care relationship (Table 2). These have included instances of physical, psychological, and sexual misbehavior. Physical misbehavior has involved aggressive and violent behavior towards patients^9,31–33,35,37,38,41,47,48,62,65^ to secure cooperation or conduct treatment safely.^37,38^ Such instances of physical violence have been reported to occur during childbirth and care facilities where patients’ capacity for cooperation was impaired due to their psychological condition or illness. Patients who had reduced levels of consciousness or were delusional were particularly likely to be victimized by nurses because they lacked the power to complain or defend themselves.^47^ Nurses have also intentionally harmed or injured patients through inappropriate treatment leading to physical injuries or infections during delivery,^39,62^ and because of harsh treatment practices in childbirth; in the most severe cases, this had resulted in new born deaths.^33,35,49^ Patients have suffered pain and discomfort because of unnecessarily frequent painful examinations by trainees or incompetent nurses,^30,33,41,48^ and nurses have also unnecessarily subjected patients to chemical restraint to perform treatment on uncooperative patients.^30,36,39,40,43^

Psychological misbehavior by nurses has included being disrespectful, verbally abusive, intimidating, and discriminatory towards patients (Table 2). Nurses have behaved in uncompassionate and uncaring ways^30,35,47,48,52,61^ by not offering comfort^30,33,34,38,48,62^ or expressing understanding, and by being insensitive to a patient’s situation and suffering.^36,37,48,62^ Nurses may have showed disrespectful behavior towards patients by insulting them,^9,35,38,40,48^ making inappropriate facial expressions,^30,33^ or mocking them in front of others.^40,41,47^ Nurses verbal abuse has frightened patients^9,30,38,41^ by shouting, using rude language,^30,33,34,36–39,42,62,65^ or silencing them with sarcastic and inappropriate comments.^37,44^ Additionally, some nurses have intimidated patients with threats of violence^9,30,39,48,61^ and punishment^40,48^ for not following care instructions.^35,36,48^ Other nurses have threatened to withhold care from patients or left patients unattended because they were crying or shouting in pain.^30,33,36^ Nurses have also treated patients unequally,^48,61^ discriminating on grounds of age, ethnicity, religion, disease, or social economic status.^35–37,47,48^ Some nurses have shown a lack of respect for patients as individuals^36,37,61^ and judged^33,48^ or blamed patients because of their own misbehavior^33,36–38,42,48^ or patients’ inability to do what nurses required.^38,48,62^

One type of violations of patient integrity has been sexual misbehavior.^46,65^ During patient care, this may have involved violating patients’ personal boundaries by touching and acting in physically inappropriate ways towards patients during nursing treatments.^39,44,54^ Patients have experienced psychological sexual violations by nurses who have used obscene language or harassed them sexually.^39^ Other forms of sexual misbehavior by nurses have included sexual abuse by petting and forced intercourse.^44,54^ Nurses have crossed their professional boundaries by having inappropriate relationships with patients either consensual sex or affairs. Relationships between a nurse and a patient may have involved kissing, hugging, sharing gifts, and non-work-related involvement with sexual contact. Most cases of this type have involved male nurses, although some female nurses have crossed professional boundaries by having an affair with a patient.^46^ Nurses’ sexual misbehavior has been reported to occur in psychiatric care facilities^44,52^ or in the home care of patients with disabilities.^39,47^

### Consequences of unprofessional behavior in patient care

Nurses’ unprofessional behavior in patient care causes patient dissatisfaction and poses risks to patient safety (Table 3).

**Table 3.** Consequences of unprofessional behavior by nurses in patient care.

Main category	Sub-category
Patient dissatisfaction	Negative treatment experiences^6,9,30–32,34,36–39,49,59,62^
	Nurses’ uncooperative behavior^6,30,31,33,34,36–39,48,49,61,62,64^
	Difficulties in obtaining care^30,33,36,61^
Risking patient safety	Adverse events and errors^6,8,32,48–51,56–60,62,65^
	Unnecessary suffering^6,22,30–34,36,48,61^
	Delayed treatment^6,30,31,39,49,50,59,61,62^
	Patient mortality^31,49,56,59,62^
	Physical or emotional injury^30,33,36,37,39,48,53,54,62^

Unprofessional behavior by nurses has led to patient dissatisfaction, which can in extreme cases leave patients afraid and unwilling to return to the hospital in the future.^9,34,37,38^ Patient dissatisfaction has resulted from negative experiences in care, nurses’ uncooperative behavior, and difficulties in obtaining care. In individual^31,36,38,39^ and group discussions,^30,38^ patients have described negative treatment experiences^6,30,32,49,59,62^ that adversely affected their mental wellbeing and ability to care for themselves.^39,48,49^ Nurses uncooperative behavior has led to patients’ negative feelings during treatment, sometimes to the point of crying because of nurses’ unkind and harsh behavior.^6,31,33,34,48,49^ Fear of nurses’ actions^36–38,62^ has caused distress^30,33,39^ as well as feelings of vulnerability and being trapped.^39^ They have also felt ignored because of nurses’ unwillingness to listen^36,64^ or meet their needs.^49,61^ Difficulties in obtaining care or delays in treatment have caused patients to fear potential consequences,^36^ and some have refused treatment because of fear^61^ or loss of confidence in their healthcare.^30,61^ Shame and shyness caused by being insulted and humiliated by nurses reduced patients’ motivation to speak to health providers.^33,36^

Unprofessional behavior by nurses has created risks to patient safety and reduced care quality,^8,48–50^ increasing the likelihood of adverse events and errors.^6,8,48,49,51,56–59,62^ In addition, problems in the work community caused by bullying co-worker and creating poor staff relationships have weakened team collaboration and communication. This, in turn, also has increased the likelihood of adverse events and errors.^32,50,51,56–60,65^ As a result of nurses’ unprofessional behavior, patients have also experienced unnecessary suffering by inadequate care^6,22,30–33,48^ and inability to obtain required help.^34,36,61^ Some patients have described experiencing unnecessary pain and complications after procedures performed by unsupervised trainees.^30,36,48^ Slow or delayed treatment^49,50,61,62^ has jeopardized patients’ health,^30,49,59^ caused avoidable complications,^6,35,36,49^ and exposed them to physical injury.^31,39,49,59^ In extreme cases, delayed or incompetent treatment has even caused patient mortality.^31,49,56,59,62^ Some patients have been physically or emotionally injured by nurses physical and verbal violence, causing patients to experience physical and psychological pain, helplessness, and loss of autonomy.^30,33,36,37,39,48,62^ In addition, nurses’ sexual misconduct^53^ has left long-lasting negative effects on the patient’s mental health.^54^

### Reasons for nurses’ unprofessional behavior in patient care

Unprofessional behavior by nurses has been linked to personal issues, professional incompetence, and problems in the work environment and work community (Table 4).

**Table 4.** Reasons for nurses’ unprofessional behavior.

Main category	Sub-category
Personal issues	Loss of passion to work^46,47,51,54,55,65^
	Personal problems^46,47,51,54,55,65^
	Personality traits^36,51,56,58,59^
	Drug misuse or addiction^51,55^
	Traumatic experiences in past^54^
Professional incompetence	Insufficient training^29,33,35,47,48,51^
	Lack of clinical knowledge^29,33,35,47,48,51^
	Weak professional identity^6,32,47,48^
Work conditions	Stressful work^9,34,41,47,48^
	Heavy workload^6,8,9,29,32–34,41,47,48,51,58,59,66^
	Understaffing^6,8,9,29,32–34,41,47,48,51,58,59,66^
	Interruptions and time delays^49,51,58^
	Challenging patients^12,33,34,47,56,59,60,63^
	Lack of equipment and medication^33,34,47,48,55^
Unclear organizational structures	Lack of support by leaders^6,9,29,32,41,47,50^
	Lack of understanding organizational rules^47,48,51,59^
	Twisted work culture^47,48^

Personal issues have been identified as a major cause of unprofessional behavior in patient care (Table 4). Particularly, impactful issues have included a loss of passion for nursing and personal problems such as physical or mental illness^46,47,51,65^ that reduce the nurse’s ability to perform effectively.^51,54,55^ Personality traits such as a disruptive personality,^56,58^ or deep seated values, have invoked to justify negative behavior.^36,51,59^ Drug misuse or addiction^55^ has also been linked to unprofessional behavior by individual nurses. In some cases, nurses have fallen into opioid addiction because of stress and heavy workloads.^51,55^ The unprofessional behavior of some nurses may have been caused by traumatic experiences in childhood.^54^

Professional incompetence has been found to be one reason for unprofessional behavior by nurses (Table 4). Professional incompetence has rooted insufficient training in relation to their work duties or a lack of clinical knowledge, because of unfinished training. This has worsened the quality of the nurses’ work and increased the risk of errors.^29,33,35,47,48,51^ In addition, nurses have struggled to maintain a strong professional identity because of exhaustion in their work, which in turn undermines the quality of their task performance.^6,32,47,48^

Work conditions have found to be related to unprofessional nursing behavior in patient care (Table 4). Nurses have found their work stressful,^9,34,41,48^ not least because they have many responsibilities.^41,47,48^ In addition, they may have reported to have heavy workloads because of understaffing and high patient-to-nurse ratios.^6,8,9,29,32–34,41,47,48,51,58,59,66^ Heavy workloads have caused interruptions and delays, making it difficult for nurses to perform their job properly.^49,51,58^ Nurses’ professional competence has also been weakened by having to perform an excessively wide range of tasks and workplace rotation.^32,47^ These issues may have been exacerbated by uneven distribution of work and responsibility for patients, or by delays in treatment caused by colleagues’ failure to provide aid.^12,56,63^ Having to treat uncooperative and challenging patients has further increased nurses’ stress and frustration,^33,34,47^ leading to a loss of focus on patient care.^56,59,60^ A lack of equipment and medication has also made nurses’ work more difficult.^33,34,47,48,55^

Nurses’ unprofessional behavior has been linked to unclear organizational structures. This has referred to lack of support from leaders. Nurses have not received feedback on their work and opportunities for career development, and they have experienced unfair treatment by leaders.^6,9,29,32,41,47,50^ This has caused dissatisfaction and reduced nurses’ motivation at work.^6,9,29,32,41,47,50^ Nurses have lacked an understanding of organizational rules, exposing them to unprofessional behavior.^47,48,51^ Nurses have not been aware of organizational goals and expectations, which has contributed to incompetent performance in nursing care.^51,59^ Twisted work culture has become embedded within the working community as a whole. This has led nurses to accept violence in care situations; this unsurprisingly has poor effects on patient outcomes.^47,48^

## Discussion

Our research data revealed that the unprofessional behavior of nurses has been described using several terms related to neglect, abuse, unethical behavior, and misbehavior. We identified different types of unprofessional behavior by nurses in patient care, including neglecting nursing duties, betraying professional confidence and violating patient integrity. The data showed that nurses’ unprofessional behavior leads to patient dissatisfaction and risk endangers patient safety. Our results suggest that the personal issues, the working environment, and organizational factors such as resource availability and management actions are the main underlying causes of unprofessional nursing behavior.

Our findings show that the term “nurses’ unprofessional behavior” refers to a wide variety of negative conduct or actions that violate the norms of good nursing practice. Professional misconduct by nurses was usually associated with actions such as theft, drug misuse, and sexual misconduct towards patients. However, the distinction between these two terms is not entirely clear, as they are also used in similar contexts, such as when describing unintentional unprofessional behavior or the delivery of generally poor care. The umbrella term of unprofessional behavior covers a broad spectrum of inadequate, unethical, and negligent behaviors in patient care, all of which hinder safe treatment practices and risk harming patient outcomes and nurses’ wellbeing.^3,14,22^ Because unprofessional behavior is a highly multidimensional term, it is challenging to recognize and report.^5,67^ While the inclusion of many different behaviors under the umbrella could in theory facilitate its recognition, in practice it can lead to confusion and misunderstanding.^16,19^ Further research is needed to achieve conceptual clarity, in order to support both future research and clinical discussion. This makes it essential to explore different forms of unprofessional behavior in detail because effective prevention requires a good understanding of the types of behavior that are being targeted.

Based on our results, neglect of nursing tasks was prevalent in studies, particularly in hospitals across different wards, in emergency departments, and during childbirth. In most cases, the reason was time pressure and a high patient-to nurse ratio, which left nurses unable to monitor all patients adequately or respond to their needs quickly enough. It can be highlighted that nurses are expected to respect ethical and moral norms when performing their profession, but they face heavy workloads and complex situations that force them to make choices and prioritize. As a result, some nurses become fatigued and lose the passion that helps uphold professional ethics.^2,5,6,49^ Unprofessional behavior by nurses may reflect wider problems in the healthcare organization rather than individual failings, and that such problems can have far-reaching impacts on both nurses’ wellbeing and the organizational culture.^10,16,21^ These findings show that disempowering and overcrowded health systems create concerns about treatment safety^9,34,37,38^ and can lead to high rates of dissatisfaction, burnout, and turnover among nurses, all of which adversely affect quality of care.^15–17,19^ To effectively manage nurses’ unprofessional behavior, it is necessary to address both nurses’ ethical skills and workplace conditions and staffing^1,68,69^ so as to provide safe conditions for both nurses and patients.^1,68^ More studies are needed to identify the best ways for organizations to detect unprofessional behavior in its early stages and intervene effectively to prevent harm to patient safety and nurses’ careers.

Our data included several studies on violence against women during childbirth. The actions of nurses were often inhumane, disrespectful, and even dangerous, causing harm to mothers or to newborns. Psychological and physical violence against women during childbirth has been identified as a global concern, particularly in developing countries.^70,71^ Based on previous studies, patients with low socio-economic status have been found to be more likely exposed for nurses behavior during childbirth.^31,36,37,62^ The most critical issue is that nurses have not recognized the harmfulness of their behavior, instead they have acted in what they believe to be the best interest of the mother and baby.^70,71^ This highlights the importance of supporting nurses in taking ethical approach that respects the human dignity of each patient.

It is noteworthy that the vast majority of studies were conducted from nurses’ perspective. It is crucial to study the patients’ point of view in order to identify hidden issues and possible unprofessional behavior by nurses towards patients in situations without witnesses. Learning from various databases, such as patient complaints and incident reports, should be used to improve our understanding of these hidden ethical issues.^3,8,60^ In addition, future research should be expanded to cover a wide range of specialties. Previous studies in this review have been carried out in some hospital departments, psychiatric wards, and home care settings, highlighting the need to consider nurses working with other vulnerable groups, such as patients in pediatric and elderly care settings.^15,16,72,73^

### Strength and limitations

The main limitations of this study relate to its research method and the diversity of the research data. All collation and charting of the data was done by a single researcher, but the summarizing and reporting of the results were performed by the research team collectively. During this process, the goals and implementation of the research process were discussed, as were any disagreements that arose, ultimately enabling a consensus to be reached.^69^ These research findings do not provide strong evidence of the phenomenon despite the fact that a large number of relevant articles were retrieved. The research data focused primarily on the hospital environment and did not comprehensively cover the full range of nursing settings, such as pediatrics, psychiatry, and home care. Only articles written in English were considered eligible for inclusion in the review because of the cost and the time needed to translate publications in other languages. As a result, some relevant articles may have been overlooked. A deeper analysis would not have been appropriate due to the diversity of research settings examined in the reviewed publications and their broad range of outcomes. A key strength of this scoping review is that it transparently maps and summarizes current knowledge of the field, presenting this knowledge in a format that is readily accessible to practitioners.^26^

## Conclusion

The results presented herein show that unprofessional behavior by nurses in patient care is a multifaceted phenomenon covering different types of actions by nurses. Effective identification and prevention of unprofessional behavior require a conceptual clarity and expanding research to cover healthcare facilities, such as those for older people and pediatric care. Adopting patients’ point of view, using multiple research methods such as analyses may help us to better understand the hidden ethical issues in care. Identifying nurses’ unprofessional behavior is challenging and requires careful consideration of their working conditions and ethical skills in order to ensure patient safety and avoid undue harm to the nursing profession and the careers of the nurses involved. The core goal is to ensure that nurses have skills and knowledge to provide dignified care to each patient.

## Appendix 1 Research articles included in the review

**Table:**

Author(s)/Country/Year	Aim(s)	Methods	Concepts
Qualitative research articles			
Balde et al. 2017. Guinea.^37^	To explore the perceptions and experiences of mistreatment during childbirth, from the perspectives of women and service providers.	Qualitative interviews and focus group discussions. Data: 64 interviews and 8 focus group discussion. 40 women of reproductive age, 13 midwives, 5 doctors, and 6 administrators.	Mistreatment and abuse
Bohren et al. 2017. Nigeria.^36^	To explore women and providers’ experiences and perceptions of mistreatment during childbirth.	Qualitative interviews and focus group discussions. Data: 41 women in reproductive age and 4 focus group discussions. 17 nurses/midwifes, 17 doctors, ,and 9 administrations.	Mistreatment and abuse
Dey et al. 2017. India.^33^	To assess the discordance between self-reported and observed measures of mistreatment of women during childbirth in public health facilities in Uttar Pradesh, India.	Cross-sectional data were collected through direct observation of deliveries and follow-up interviews with women. Data: 875 women delivering in 81 public health facilities.	Mistreatment and abuse
Dixon 2013. Great Britain.^41^	To uncover and critically examine hidden assumptions of nurses’ unethical conduct arising from inquiries in Nurses Tribunal.	A qualitative study with a post-structural theoretical framework and discourse analysis. Data: Complete transcripts of five Tribunal inquiries from the period 1998 to 2003.	Unethical conduct
Gebremichael et al. 2018. Ethiopia.^29^	To describe women’s experience of disrespect and abuse during labor.	Qualitative focus group discussions with semi-structured discussion guide. Data: Eight focus groups, total of 62 women.	Disrespectful and abusive care
Ghobadi et al. 2024. Iran.^49^	To explain the factors influencing professional misconduct by nurses.	Qualitative semi-structured interviews. Data: 19 nurses.	Professional misconduct
Haseli et al. 2024. Sweden.^51^	To explore the threats to women’s dignity during childbirth.	Qualitative interviews. Data: 32 midwifery students.	Professional misconduct
Hassouneh-Phillips & McNeff 2004. USA.^39^	Investigate care-related abuse and neglect in the lives of women with SCI.	Qualitative interviews. Data: 13 women with SCI.	Care-related abuse and neglect
Kerber et al. 2015. USA.^60^	To describe new nurses’ perceptions of incivility in the workplace and to explore the impact of incivility on new nurses and patients.	Qualitative online questionnaire with open-ended questions. Data: 79 new graduate nurses	Incivility
Mapumulo et al. 2021. South Africa.^31^	To describe informal working women’s experiences of disrespectful care in health facilities during pregnancy, childbirth, and caring for young child.	Qualitative interviews. Data: Three participatory focus group discussions with 24 women.	Disrespectful and abusive care
Maya et al. 2018. Ghana.^32^	To explore women’s perspectives on mistreatment during facility-based childbirth.	Focus group discussions and in-depth interviews. Data: 39 in-depth interviews, 10 focus group discussions involving 110 delivering women.	Mistreatment and abuse
Mayra et al. 2022. India.^30^	To investigate the causes of mistreatment of women during childbirth.	Qualitative research. Data: 34 in-depth semi-structured interviews of midwifery and nursing leaders, who have years of care experience.	Disrespect and abuse
Mselle et al. 2019. Tanzania.^34^	To describe the experience of mothers and fathers in relation to treatment and mistreatment during childbirth in Tanzania.	Qualitative interviews. Data: 12 participants, four focus group discussions with mothers (6) and fathers (6).	Mistreatment and abuse
Oluoch-Aridi et al. 2018. Kenya.^35^	To explore the experiences and perceptions of both female patients and healthcare workers regarding mistreatment during childbirth.	Qualitative research with focus group discussions and in-depth interviews. Data: 46 in-depth interviews and 15 focus group discussions with delivered women. 20 in-depth interviews and observations with healthcare workers.	Mistreatment and abuse
Pavithra et al. 2022. Australia.^8^	To develop understanding of hospital staff experiences of unprofessional behaviors and their impact on staff and patients	Qualitative survey with two open-ended questions. Data: 5178 survey respondents from staff members in eight hospitals	Unprofessional behavior (negative behaviors)
Pugh 2011. Australia.^47^	To examine the experiences of registered nurses who were reported to authority for an allegation of unprofessional conduct.	Grounded theory study. In-depth interviews. Data: 21 registered nurses.	Unprofessional conduct
Pugh 2009. Australia.^46^	To develop a grounded theory study of how nurses deal with an allegation of unprofessional conduct.	In-depth qualitative interviews. Data: 21 registered nurses	Unprofessional conduct
Samson-Mojares et al. 2019. USA.^61^	To acquire an understanding and develop a theory to address incivility in nursing.	Semi-structured qualitative interviews. Data: 25 registered nurses.	Incivility
Shimoda et al. 2018. Tanzania.^28^	To describe respectful and disrespectful care in labor carried out by midwifes.	Descriptive qualitative study with naturalistic observations. Data: 14 midwifes who took care of 24 labors.	Disrespectful and abusive care
Vagharseyyedin 2016. Iran.^12^	To report nurses’ perspectives concerning workplace mistreatment.	Qualitative interviews. Data: 15 nurses.	Unethical conduct
Varaei et al. 2024. Iran.^50^	To explain the experiences of nurses regarding the outcomes of professional misconduct.	Semi-structured qualitative interviews. Data: 22 nurses and nursing managers.	Professional misconduct
Wang et al. 2022. China.^6^	To explore nurses’ ethical behavior and what contributes to unethical behavior.	Qualitative focus group and semi-structured individual interviews. Data: 30 nurses.	Unethical behavior
Woith et al. 2017. USA.^62^	To explore homeless people’s perceptions of their interactions with nurses.	Qualitative interviews with Husserl`s phenomenological approach Data: 15 homeless adults.	Incivility
Quantitative research articles			
AbuDagga et al. 2019. USA.^53^	To examine nurse sexual misconduct-related reports and compare them with reports for other types of offences.	Cross-sectional study. Descriptive analyses of reports in the National Practitioner Data Bank both licensure and malpractice-payment reports. Data: 882 sexual misconduct-related reports.	Sexual misconduct
Alzyoud et al. 2023. USA.^70^	To understand the factors influencing nurses’ and midwifes’ disrespectful and abusive behavior during labor.	Cross-sectional study design, online electronic survey. Data: 231 nurses and midwifes.	Disrespect and abuse
Asefa et al. 2018. Ethiopia.^9^	To present service providers experiences of disrespectful and abusive practices during childbirth.	Quantitative questionnaire. Data: 57 health professionals.	Disrespectful and abusive care
Bachmann et al. 2000. Switzerland.^52^	To determine the frequency of nurse–patient sexual relationships and their prominent characteristics and the nurses’ attitudes towards sexual contacts.	Quantitative questionnaire with 35 items. Data: 279 nurses in two psychiatric hospitals.	Sexual misconduct
Chiarella & Adrian 2014. Australia.^43^	To explore the spectrum of boundary violations in the nurse-patient relationship.	Document analyses of the complaints and disciplinary decisions related to registered nurses and registered midwifes boundary violations. Data = 29 documents.	Professional misconduct
Erdil & Kormaz 2009. Turkey.^40^	To determine ethical problems in healthcare observed by nursing students.	Questionnaire. Data: 153 nursing students.	Unethical conduct
Kunyk et al. 2016. Canada.^54^	Explore opioid addiction in nursing and opioid-related misconduct.	Critical discourse analysis. Publicly available media text and legal documentation of two cases of opioid-addicted nurses.	Opioid addiction
Larry & Veltman 2007. USA.^59^	To determine the frequency of disruptive behavior in labor and delivery units and its effect on work performance and adverse events.	Quantitative questionnaire. Data: 56 nurse managers from labor and delivery unit.	Disruptive behavior
Malmedal et al. 2009. Norway.^38^	To describe the frequency and types of inadequate care in nursing homes committed by staff.	Quantitative questionnaire survey in 16 nursing homes. Data: 616 nurses.	Inadequate care and neglect
Mauritz et al. 2016. Netherlands.^44^	Investigate homecare nurses experiences of professional misconduct, reporting it and preventing professional misconduct.	Quantitative questionnaire. Data: 259 nurses and certified nurse assistants.	Professional misconduct
Nauman et al. 2022. Pakistan.^63^	To investigate how patient incivility is linked with nurses’ unethical behavior and organizational citizenship behavior.	Quantitative questionnaire. Data: two questionnaires with a total of 373 and 351 received responses.	Unethical behavior
Rosenstein, & Naylor 2012. USA.^55^	To assess disruptive behaviors and staff relationships in the ED.	Quantitative electronic survey. Data: 237 nurses, 133 other health workers from 20 individual EDs.	Disruptive behavior
Rosenstein & O’Daniel 2008. USA.^57^	To assess the effect of disruptive behaviors on communication and collaboration and impact on patient care.	Quantitative survey in 102 hospitals. Data: 2846 nurses, 1684 physician, or other health worker.	Disruptive behavior
Rosenstein & O’Daniel 2005. USA.^56^	To examine the disruptive behavior of both physicians and nurses, as well as both groups’ and administrators’ perceptions of its effects on providers and its impact on clinical outcomes.	Quantitative survey. Data: 1500 participants in 50 hospitals across country.	Disruptive behavior
Mixed-method research articles			
Addison & Luparell 2014. USA.^58^	To explore rural nurses’ perceptions of disruptive behavior and its influence on patient safety, patient outcomes, and disciplinary relationships.	Electronic questionnaire with quantitative and qualitative questions. Data: 57 nurses in two central hospitals.	Disruptive behavior
Fida et al. 2016. Italy.^42^	To develop and validate nursing moral disengagement scale and investigate association between moral disengagement and counterproductive and citizenship behavior.	Qualitative and quantitative combining a cross-validation approach and a structural equation model. Data: Qualitative study, 60 nurses. Quantitative study, 434 nurses	Moral disengagement
Searle & Rice. 2021. United Kingdom.^48^	To explore similarities and differences regarding misconduct of three profession groups: doctors, nurses, and midwifes.	Mixed-method study. Quantitative and qualitative document analysis. Quantitative data: 6714 documents of fitness to practice incidents involving 16 healthcare professions were analyzed. Qualitative data: 72 incidents (13 doctors, 38 nurses and midwifes, and 21 allied health professions) were analyzed more deeply.	Professional misconduct

## References

[1] Soltanian M Molazem Z Mohammadi E , et al. Iranian nurses’ experiences on obstacles of safe drug administration: a qualitative study. Glob J Health Sci 2016; 8: 56009. 10.5539/gjhs.v8n10p88 27302450

[2] Fagbenro DA . Role ambiguity and organizational justice as the predictors of unethical behavior among nurses. J Client Cent Nurs Care 2019; 5: 81–86. 10.32598/jccnc.5.2.81

[3] Westbrook J Sunderland N Atkinson V , et al. Endemic unprofessional behaviour in health care: the mandate for a change in approach. Med J Aust 2018; 209: 380–381. 30376656 10.5694/mja17.01261

[4] Alshehry AS Alquwez N Almazan J , et al. Influence of workplace incivility on the quality of nursing care. J Clin Nurs 2019; 28: 4582–4594. 10.1111/jocn.15051 31494996

[5] Orathai P Prapaipanich W Arpanantikul M , et al. Development and psychometric evaluation of the ethical behavior for Thai nurses scale. Front Nurs 2022; 9: 275–284. 10.2478/fon-2022-0034

[6] Wang S Jiang Z Zhang Z , et al. The status of ethical behaviour in clinical nursing in three Chinese hospitals: a qualitative interview study. J Nurs Manag 2022; 30: 2424–2433. 10.1111/jonm.13810 36121743

[7] Westbrook J Sunderland N Li L , et al. The prevalence and impact of unprofessional behaviour among hospital workers: a survey in seven Australian hospitals. Med J Aust 2021; 214: 31–37. 10.5694/mja2.50849 33174226

[8] Pavithra A Sunderland N Callen J , et al. Unprofessional behaviours experienced by hospital staff: qualitative analysis of narrative comments in a longitudinal survey across seven hospitals in Australia. BMC Health Serv Res 2022; 22: 410. 10.1186/s12913-022-07763-3 35351097 PMC8962235

[9] Asefa A Bekele D Morgan A , et al. Service providers’ experiences of disrespectful and abusive behavior towards women during facility based childbirth in Addis Ababa, Ethiopia. Reprod Health 2018; 15: 4. 10.1186/s12978-017-0449-4 29304814 PMC5756390

[10] Bloom EM . Horizontal violence among nurses: experiences, responses, and job performance. Nurs Forum 2019; 54: 77–83. 10.1111/nuf.12300 30332520

[11] Papinaho O Häggman-Laitila A Kangasniemi M . Unprofessional conduct by nurses: a document analysis of disciplinary decisions. Nurs Ethics 2022; 29: 131–144. 10.1177/09697330211015289 34583555 PMC8866744

[12] Vagharseyyedin SA . Nurses’ perspectives on workplace mistreatment: a qualitative study. Nurs Health Sci 2016; 18: 70–78. 10.1111/nhs.12236 26573988

[13] World Health Organization . Building better together roadmap to guide implementation of the Global Strategic Directions for nursing and midwifery in the WHO European Region. WHO Regional Office for Europe, 2021.

[14] Abolfazl Vagharseyyedin S . Workplace incivility: a concept analysis. Contemp Nurse 2015; 50: 115–125. 10.1080/10376178.2015.1010262 26213258

[15] D’ambra AM Andrews DR . Incivility, retention and new graduate nurses: an integrated review of the literature. J Nurs Manag 2014; 22: 735–742. 10.1111/jonm.12060 23927565

[16] Bambi S Foà C De Felippis C , et al. Workplace incivility, lateral violence and bullying among nurses. A review about their prevalence and related factors. Acta Biomed 2018; 89: 51–79. 10.23750/abm.v89i6-S.7461 30038204 PMC6357596

[17] Saxton R Hines T Enriquez M . The negative impact of nurse-physician disruptive behavior on patient safety: a review of the literature. J Patient Saf 2009; 5: 180–183. 10.1097/PTS.0b013e3181b4c5d7 19927052

[18] Manfrin-Ledet L Porche D Eymard A . Professional boundary violations. A literature review. Home Healthc Now 2015; 33: 326–332. 10.1097/NHH.0000000000000249 26034824

[19] Ditmer D . A safe environment for nurses and patients: halting horizontal violence. J Nurs Regul 2010; 1: 9–14. 10.1016/s2155-8256(15)30327-6

[20] Hulme S Hughes CE Nielsen S . What factors contributed to the misconduct of health practitioners? An analysis of Australian cases involving the diversion and supply of pharmaceutical drugs for non‐medical use between 2010 and 2016. Drug Alcohol Rev 2019; 38: 366–376. 10.1111/dar.12918 30887600

[21] Parizad N Hassankhani H Rahmani A , et al. Nurses’ experiences of unprofessional behaviors in the emergency department: a qualitative study. Nurs Health Sci 2018; 20: 54–59. 10.1111/nhs.12386 29154396

[22] Zaghini F Fida R Kangasniemi M , et al. What is behind counterproductive work behaviors in the nursing profession? A systematic review. J Clin Res Bioeth 2016; 7: 1000277.

[23] Al Abrawi S . A concept analysis of misconduct: application to nursing education. Nurs Ethics 2024; 31: 89–100. 10.1177/09697330231183080 37403622

[24] Laschinger HKS . Impact of workplace mistreatment on patient safety risk and nurse-assessed patient outcomes. J Nurs Adm 2014; 44: 284–290. 10.1097/NNA.0000000000000068 24759201

[25] Ekpenyong MS Nyashanu M Ibrahim A , et al. Perceived barriers to whistle blowing in healthcare amongst healthcare professionals: an integrative review. Int J Human Rights Healthc 2020; 14: 10–27. 10.1108/ijhrh-08-2020-0064

[26] Arksey H O’Malley L . Scoping studies: towards a methodological framework. Int J Soc Res Methodol 2005; 8: 19–32. 10.1080/1364557032000119616

[27] Munn Z Peters MDJ Stern C , et al. Systematic review or scoping review? Guidance for authors when choosing between a systematic or scoping review approach. BMC Med Res Methodol 2018; 18: 143. 10.1186/s12874-018-0611-x 30453902 PMC6245623

[28] Peters MDJ Marnie C Tricco AC , et al. Updated methodological guidance for the conduct of scoping reviews. JBI Evid Synth 2020; 18: 2119–2126. 10.11124/JBIES-20-00167 33038124

[29] Shimoda K Horiuchi S Leshabari S , et al. Midwives’ respect and disrespect of women during facility-based childbirth in urban Tanzania: a qualitative study. Reprod Health 2018; 15: 8. 10.1186/s12978-017-0447-6 29321051 PMC5763614

[30] Gebremichael MW Worku A Medhanyie AA , et al. Women suffer more from disrespectful and abusive care than from the labour pain itself: a qualitative study from women’s perspective. BMC Pregnancy Childbirth 2018; 18: 392. 10.1186/s12884-018-2026-4 30286734 PMC6172829

[31] Mayra K Matthews Z Padmadas SS . Why do some health care providers disrespect and abuse women during childbirth in India? Women Birth 2022; 35: e49–e59. 10.1016/j.wombi.2021.02.003 33678563

[32] Mapumulo S Haskins L Luthuli S , et al. Health workers’ disrespectful and abusive behaviour towards women during labour and delivery: a qualitative study in Durban, South Africa. PLoS One 2021; 16: e0261204. 10.1371/journal.pone.0261204 34905562 PMC8670673

[33] Dey A Shakya HB Chandurkar D , et al. Discordance in self-report and observation data on mistreatment of women by providers during childbirth in Uttar Pradesh, India. Reprod Health 2017; 14: 149. 10.1186/s12978-017-0409-z 29141640 PMC5688759

[34] Mselle LT Kohi TW Dol J . Humanizing birth in Tanzania: a qualitative study on the (mis) treatment of women during childbirth from the perspective of mothers and fathers. BMC Pregnancy Childbirth 2019; 19: 231. 10.1186/s12884-019-2385-5 31277609 PMC6612108

[35] Oluoch-Aridi J Smith-Oka V Milan E , et al. Exploring mistreatment of women during childbirth in a peri-urban setting in Kenya: experiences and perceptions of women and healthcare providers. Reprod Health 2018; 15: 209. 10.1186/s12978-018-0643-z 30558618 PMC6296108

[36] Bohren MA Vogel JP Tunçalp Ö , et al. Mistreatment of women during childbirth in Abuja, Nigeria: a qualitative study on perceptions and experiences of women and healthcare providers. Reprod Health 2017; 14: 9. 10.1186/s12978-016-0265-2 28095911 PMC5240205

[37] Balde MD Diallo BA Bangoura A , et al. Perceptions and experiences of the mistreatment of women during childbirth in health facilities in Guinea: a qualitative study with women and service providers. Reprod Health 2017; 14: 3. 10.1186/s12978-016-0266-1 28077145 PMC5225581

[38] Nauman S Malik SZ Saleem F . The slippery slope effect of patient incivility: unleashing the roles of surface acting and receiving help in employees’ unethical behavior and organizational citizenship behavior. Int J Hum Resour Manag 2022; 34: 3491–3519. 10.1080/09585192.2022.2129418

[39] Hassouneh-Phillips DS McNeff E . Understanding care-related abuse and neglect in the lives of women with SCI. Sci Nurs 2004; 21: 75–81. 15553077

[40] Erdil F Korkmaz F . Ethical problems observed by student nurses. Nurs Ethics 2009; 16: 589–598. 10.1177/0969733009106651 19671645

[41] Malmedal W Ingebrigtsen O Saveman B . Inadequate care in Norwegian nursing homes – as reported by nursing staff. Scand J Caring Sci 2009; 23: 231–242. 10.1111/j.1471-6712.2008.00611.x 19662673

[42] Dixon KA . Unethical conduct by the nurse: a critical discourse analysis of Nurses Tribunal inquiries. Nurs Ethics 2013; 20: 578–588. 10.1177/0969733012468465 23378542

[43] Fida R Tramontano C Paciello M , et al. Nurse moral disengagement. Nurs Ethics 2016; 23: 547–564. 10.1177/0969733015574924 25908639

[44] Chiarella M Adrian A . Boundary violations, gender and the nature of nursing work. Nurs Ethics 2014; 21: 267–277. 10.1177/0969733013493214 23981809

[45] Pugh D . The phoenix process: a substantive theory about allegations of unprofessional conduct. J Adv Nurs 2009; 65: 2027–2037. 10.1111/j.1365-2648.2009.05038.x 19686404

[46] Searle RH Rice C . Making an impact in healthcare contexts: insights from a mixed-methods study of professional misconduct. Eur J Work Organ Psychol 2021; 30: 470–481. 10.1080/1359432x.2020.1850520

[47] Maurits EEM De Veer AJE Groenewegen PP , et al. Dealing with professional misconduct by colleagues in home care: a nationwide survey among nursing staff. BMC Nurs 2016; 15: 59. 10.1186/s12912-016-0182-2 27777510 PMC5062941

[48] Millbank J . Serious misconduct of health professionals in disciplinary tribunals under the national law 2010–17. Aust Health Rev 2020; 44: 190–199. 10.1071/AH18239 31671287

[49] Ghobadi A Sayadi L Nayeri ND , et al. The nurses’ perception of the factors influencing professional misconduct: a qualitative study. Nurs Ethics 2024; 31: 281–295. 10.1177/09697330231184469 37599451

[50] Varaei S Nayeri ND Sayadi L , et al. Outcomes of professional misconduct by nurses: a qualitative study. BMC Nurs 2024; 23: 200. 10.1186/s12912-024-01859-3 38528519 PMC10962125

[51] Haseli A Khosravi S Hajimirzaie SS , et al. Midwifery students’ experiences: violations of dignity during childbirth. Nurs Ethics 2024; 31: 296–310. 10.1177/09697330231197703 37650382 PMC11181724

[52] Bachmann KM Bossi J Moggi F , et al. Nurse–patient sexual contact in psychiatric hospitals. Arch Sex Behav 2000; 29: 335–347. 10.1023/a:1001914303435 10948723

[53] AbuDagga A Wolfe SM Carome M , et al. Crossing the line: sexual misconduct by nurses reported to the National practitioner data bank. Public Health Nurs 2019; 36: 109–117. 10.1111/phn.12567 30556923 PMC7380059

[54] Kunyk D Milner M Overend A . Disciplining virtue: investigating the discourses of opioid addiction in nursing. Nurs Inq 2016; 23: 315–326. 10.1111/nin.12144 27605201

[55] Longo J . Combating disruptive behaviors: strategies to promote a healthy work environment. Online J Issues Nurs 2010; 15: 1–13. 10.3912/ojin.vol15no01man05

[56] Rosenstein AH Naylor B . Incidence and impact of physician and nurse disruptive behaviors in the emergency department. J Emerg Med 2012; 43: 139–148. 10.1016/j.jemermed.2011.01.019 21421291

[57] Rosenstein AH O’Daniel M . Disruptive behavior and clinical outcomes: perceptions of nurses and physicians. Am J Nurs 2005; 105: 54–64. 10.1097/00000446-200501000-00025 15659998

[58] Rosenstein AH O’Daniel M . A survey of the impact of disruptive behaviors and communication defects on patient safety. Joint Comm J Qual Patient Saf 2008; 34: 464–471. 10.1016/s1553-7250(08)34058-6 18714748

[59] Addison K Luparell S . Rural nurses’ perceptions of disruptive behavior and clinical outcomes: a pilot study. Online J Rural Nurs Health Care 2014; 14: 66–82. 10.14574/ojrnhc.v14i1.300

[60] Veltman LL . Disruptive behavior in obstetrics: a hidden threat to patient safety. Am J Obstet Gynecol 2007; 196: 587.e1–587.e4. 10.1016/j.ajog.2007.03.011 17547907

[61] Kerber C Woith WM Jenkins SH , et al. Perceptions of new nurses concerning incivility in the workplace. J Contin Educ Nurs 2015; 46: 522–527. 10.3928/00220124-20151020-05 26509405

[62] Maya ET Adu-Bonsaffoh K Dako-Gyeke P , et al. Women’s perspectives of mistreatment during childbirth at health facilities in Ghana: findings from a qualitative study. Reprod Health Matters 2018; 26: 70–87. 10.1080/09688080.2018.1502020 30152268

[63] Samson-Mojares RA Chin CR Colvin MK , et al. Where do you think you are? A grounded theory study of the critical factors triggering the existence and fueling the persistence of incivility in nursing. Nurs Educ Perspect 2019; 40: 133–138. 10.1097/01.NEP.0000000000000397 31008884

[64] Woith WM Kerber C Astroth KS , et al. Lessons from the homeless: civil and uncivil interactions with nurses, self-care behaviors, and barriers to care. Nurs Forum 2017; 52: 211–220. 10.1111/nuf.12191 27922178

[65] Pugh D . A fine line: the role of personal and professional vulnerability in allegations of unprofessional conduct. J Nurs Law 2011; 14: 21–31. 10.1891/1073-7472.14.1.21

[66] Laukkanen L Suhonen R Leino-Kilpi H . Solving work-related ethical problems: the activities of nurse managers. Nurs Ethics 2016; 23: 838–850. 10.1177/0969733015584966 26038376

[67] Laukkanen L Suhonen R Poikkeus T , et al. The effectiveness of the Ethics Quarter intervention on the ethical activity profile of nurse managers: a randomized controlled trial. J Nurs Manag 2022; 30: 2126–2137. 10.1111/jonm.13411 34231275

[68] Johnstone M Kanitsaki O . Processes for disciplining nurses for unprofessional conduct of a serious nature: a critique. J Adv Nurs 2005; 50: 363–371. 10.1111/j.1365-2648.2005.03401.x 15842443

[69] ALLEA - All European Academies . The European code of conduct for research integrity. , 2017.

[70] Yalley AA Jarašiūnaitė-Fedosejeva G Kömürcü-Akik B , et al. Addressing obstetric violence: a scoping review of interventions in healthcare and their impact on maternal care quality. Front Public Health 2024; 12: 1388858. 10.3389/fpubh.2024.1388858 38979044 PMC11228167

[71] Özer E ÇetiNkaya Şen Y Canli S . Evaluation of the prevalence of obstetric violence during child birth: a meta-analysis study. Aggress Violent Behav 2025; 83: 102067. 10.1016/j.avb.2025.102067

[72] Ditmer D . A safe environment for nurses and patients: halting horizontal violence. J Nurs Regul 2010; 1: 9–14. 10.1016/s2155-8256(15)30327-6

[73] Pazokian M Zagheri Tafreshi M Rassouli M . Iranian nurses’ perspectives on factors influencing medication errors. Int Nurs Rev 2014; 61: 246–254. 10.1111/inr.12086 24571495

